# Surgical Management of Zollinger‐Ellison Syndrome in Multiple Endocrine Neoplasia Type 1 an AFCE and GTE Cohort Study. (Association Francophone de Chirurgie Endocrinienne and Groupe d'étude des Tumeurs Endocrines)

**DOI:** 10.1002/wjs.70303

**Published:** 2026-03-20

**Authors:** Sébastien Gaujoux, François Pattou, Guillaume Cadiot, Mustapha Adham, Philippe Bachellier, Jean‐Pierre Bail, Robert Caiazzo, Nicolas Carrere, Philippe Chaffanjon, Sophie Deguelte, Gianluca Donatini, Bertrand Dousset, Matthieu Faron, Caroline Gronnier, Bruno Heyd, Jean‐Christophe Lifante, Jean Lubrano, Nicolas Meurisse, Eric Mirallié, Nicolas Santucci, Alain Sauvanet, Frédéric Sebag, Laurent Sulpice, Baudoin Thebault, Jean‐Jacques Tuech, Thomas Walter, Christine Binquet, Pierre Goudet

**Affiliations:** ^1^ Department of General, Visceral, and Endocrine Surgery Pitié Salpêtrière Hospital, AP‐HP Paris France; ^2^ Department of General and Endocrine Surgery Lille University Hospital Lille France; ^3^ Department of Hepato‐Gastro‐Enterology Robert‐Debré Hospital, Reims‐Champagne‐Ardennes University Reims France; ^4^ Department of Digestive Surgery, Lyon Sud Faculty of Medicine Edouard Herriot Hospital, Hospices Civils de Lyon, Claude Bernard University Lyon 1 (UCBL1) Lyon France; ^5^ Department of HPB Surgery and Liver Transplant Hautepierre Hospital, Strasbourg University Strasbourg France; ^6^ Department of Digestive Surgery Brest University Hospital Brest France; ^7^ Department of Digestive Surgery Toulouse University Hospital Toulouse France; ^8^ Department of Thoracic, Vascular and Endocrine Surgery Grenoble University Hospital Grenoble France; ^9^ Department of General and Digestive Surgery Robert‐Debré Hospital, Reims‐Champagne‐Ardennes University Reims France; ^10^ Department of General Surgery University Hospital Poitiers France; ^11^ Department of Gastrointestinal, Hepatobiliary and Endocrine Surgery Cochin Hospital, AP‐HP (Paris Public Hospitals) Paris France; ^12^ Université Paris‐Saclay, Gustave Roussy, INSERM 1018 Oncostat Team Villejuif France; ^13^ Department of Digestive Surgery Haut Lévêque University Hospital Bordeaux France; ^14^ Department of Visceral, Digestive and Oncologic Surgery University Hospital, Franche‐Comté University Besançon France; ^15^ Department of Digestive and Endocrine Surgery, Health Services and Performance Research University Hospital of Lyon Sud and EA 7425 HESPER, University Claude Bernard Lyon 1 Lyon France; ^16^ Department of Digestive Surgery University Hospital of Caen Caen cedex France; ^17^ Department of Abdominal Surgery and Transplantation Liège University Hospital University of Liège Liège Belgium; ^18^ Department of Digestive and Endocrine Surgery Nantes University Hospital Nantes France; ^19^ Department of Digestive and Endocrine Surgery Dijon University Hospital Dijon France; ^20^ Department of HPB Surgery and Liver Transplantation Hospital Beaujon, AP‐HP, & University Paris Cité Clichy France; ^21^ Department of General, Endocrine and Metabolic Surgery Conception Hospital Marseille France; ^22^ Department of HPB, Liver Transplantation and Digestive Surgery Rennes University Hospital, Inserm U1414, Rennes 1 University Rennes France; ^23^ Digestive and Endocrine Surgery Unit Orléans University Hospital Orléans France; ^24^ Department of Digestive Surgery Charles Nicolle University Hospital Rouen France; ^25^ Department of Medical Oncology Edouard Herriot Hospital, Hospices Civils de Lyon Lyon France; ^26^ Université Bourgogne Europe, CHU Dijon Bourgogne, Centre d'investigation Clinique, Module épidémiologie clinique, INSERM, CIC 1432 Dijon France; ^27^ Université Bourgogne Europe, INSERM, U1231, Centre de recherche Translationnelle en Médecine moléculaire, équipe EPICAD Dijon France

**Keywords:** duodenotomy, gastrinoma, multiple endocrine neoplasia type 1, pancreaticoduodenectomy, Zollinger‐Ellison syndrome

## Abstract

**Objective:**

To describe surgical indications, procedures and outcomes in patients operated for Zollinger‐Ellison syndrome (ZES) in multiple endocrine neoplasia type 1 (MEN1) using a large nationwide cohort.

**Background:**

Management of ZES in MEN1 remains controversial.

**Methods:**

All patients with ZES diagnosed through the MEN1 AFCE/GTE network from 1985 to 2015.

**Results:**

Among 233 ZES patients, 66 (28%) were operated for ZES‐related gastrinomas. Thirty‐three (51%) procedures aimed to remove gastrinomas and associated pancreatic neuroendocrine tumors (pNET(s)) with appropriate resection. Thirty‐two procedures (49%) aimed to remove gastrinomas alone (ZES group). Survival was decreased in patients metastatic at ZES diagnosis (*p* < 0.001). Fifteen‐year survival among non‐metastatic patients was not significantly better in operated patients (82% vs. 70%, *p* = 0.2). Perioperative mortality was nil. Metastatic lymph nodes were found in 30/42 lymphadenectomies (71%). The choice between pancreaticoduodenectomy versus duodenal focused surgery in the ZES group was associated with pre‐operative detection of adenopathies (*p* > 0.001), leading to more frequent lymphadenectomies (*p* < 0.01). Previous pancreatic surgeries (30%) may have influenced the choice of ZES procedures. Gastrin levels were more frequently normalized when the duodenum and the head of pancreas were removed versus more localized duodenal surgeries (*p* < 0.01).

**Conclusion:**

The high rate of invaded nodes in lymphadenectomies in MEN1 patients operated for ZES, the absence of operative mortality, and the decreased survival in metastatic patients are indirect arguments for surgery. Pancreaticoduodenectomy may be indicated in young and fit individuals to better control hypergastrinemia and to prevent metastatic progression in the ZES group. Gastrinoma removal is justified when associated with large pNETs.

## Graphical abstract

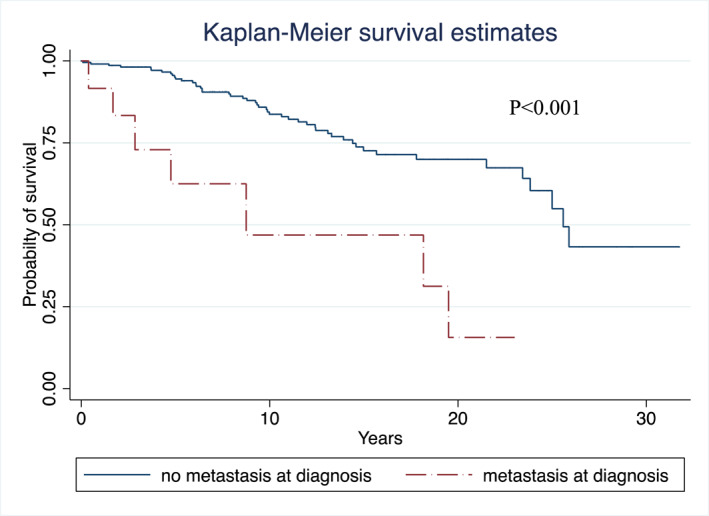

**Abbreviations**
MEN1: multiple endocrine neoplasia type 1
pNET: pancreatic neuroendocrine tumor
PPI: proton‐pump inhibitor
ZES: Zollinger‐Ellison syndrome

## 1 Introduction

Multiple endocrine neoplasia type 1 (MEN1) is an inherited disease that predisposes carriers to duodenal, pancreatic, and duodeno‐pancreatic neuroendocrine tumors (dNETs, pNETs, and dpNETs) [1, 2, 3, 4]. Gastrinomas, which are present in about 30% of patients with MEN1, are the most frequent functional dpNETs in MEN1 and are responsible for Zollinger‐Ellison syndrome (ZES) [5]. After primary hyperparathyroidism, dpNETs are the second most frequent MEN1‐associated lesion and are responsible for the majority of cancer‐related deaths in MEN1 [6, 7]. Among dpNETs, gastrinomas are also a significant cause of distant metastases and death [8, 9]. Therefore survival, metastatic prevention, possible role of surgery, surgical procedures and quality of life are current issues which are regularly debated in MEN1 publications, reviews and recommendations [2, 3, 4, 10, 11, 12, 13, 14, 15, 16, 17]. Considering that MEN1 related gastrinomas are multiple, located in the duodenum, and genetically driven, the surgical recommendations for sporadic duodenal and pancreatic gastrinomas cannot be used [15]. Previously published series are generally based on data collected in specialized centers, but there are currently no cohort studies describing real‐life surgical management for the entire MEN1‐ZES spectrum over a large territory. Therefore, the MEN1 AFCE/GTE cohort (Association Francophone de Chirurgie Endocrinienne, Groupe d’étude des Tumeurs Endocrines) was used to conduct a specific cohort study whose main objectives were: (1) to assess the characteristics of ZES patients and how their management has changed over time; (2) to understand the use of surgical procedures in clinical practice; (3) to assess the results of lymphadenectomies; (4) to analyze survival and causes of death; and (5) to measure the effects of surgery on gastrin levels.

## 2 Materials and Methods

### 2.1 Population, Definitions of MEN1 and Outcomes

The study included all patients with ZES who were part of the MEN1 cohort of the AFCE/GTE network and diagnosed between 1985 and 2015 [9]. The diagnostic criteria used for MEN1 conformed to the regularly updated international and French recommendations [2, 3, 4]. The constitution of the cohort and its legal aspects are available in the Supporting Information S1 section. ZES patients diagnosed before 1985 were excluded (lower quality imaging and less efficient anti‐acid agents). ZES patients diagnosed after 2015 were also excluded because of the limited follow‐up.

### 2.2 Diagnosis of Zollinger‐Ellison Syndrome and Surgical Indications

Patient charts mentioning ZES were reviewed for diagnosis confirmation (PG) (Supporting Information S1). Recommendations for ZES diagnosis previously required at least two out of 5 National Institute of Health (NIH) criteria [18]. Nevertheless, the lack of secreting hormone assay availability, the increasing difficulty of obtaining gastric acid output measurements, and the poor general condition of some MEN1 patients may have led clinicians to use alternative criteria after excluding atrophic gastritis [19]. ZES surgeries were carried out with curative intent and classified into two categories: (1) “ZES” surgery, i.e. in order to cure ZES only, and (2) “ZES + pNET” surgeries, that is in order to cure ZES and to remove an associated pancreatic NET (pNET ≥ 2 cm or rarely another secreting tumor such as an insulinoma or a glucagonoma). Distant metastatic disease and causes of death were also systematically collected. Tumors were categorized according to the 2017 UICC TNM classification [20]. Overall survival was compared from the date of ZES diagnosis in patients operated for ZES and in those not operated for ZES. Overall survival was also computed from the date of ZES surgery. CT‐scan results were used to assess abnormal nodes in all patients. We delineated two periods (before and after January 2000) due to major advances in CT imaging using multiple slice images (16 multi‐slice in 2001) with narrower collimation [21]. MRI imaging results were not analyzed in order to avoid methodological bias. Indeed, MRI was mainly used in second period of the study. The effect of surgery on basal gastrin levels was evaluated among ZES patients with preoperatively elevated levels. Surgery was considered effective when basal gastrin levels were normalized during the year following surgery whatever the PPI treatment, which was systematically maintained.

### 2.3 Statistical Analysis

Continuous variables are expressed as means ± SD, medians and interquartile ranges [IQR]), and/or percentages, as appropriate. Chi‐square or Fisher exact tests were used to compare differences in discrete or categorical variables, and the *t*‐test or Mann‐Whitney test was used for comparing means or medians. Overall survival probability was estimated at 5, 10 and 15 years using the Kaplan–Meier method and expressed with a 95% confidence interval (95% CI). Survival was compared between groups using the log‐rank test. All tests were two sided, and statistical significance was set at *p* < 0.05. Data were analyzed with STATA 13 statistical software (Stata Corp. 2011. Stata Statistical Software: Release 13. College Station, TX: Stata Corp LP).

## 3 Results

### 3.1 Characteristics of the Studied Cohort and Changes Over Time

Between 1985 and 2015, 233 patients were diagnosed with ZES in France. The main clinical data for operated (pancreas, duodenum and stomach) and non‐operated ZES patients are displayed in Table 1 according to the time period (< 2000 vs. ≥ 2000). Despite changes in clinical presentation and in criteria for diagnosis, age at ZES diagnosis remained stable. Fifty‐nine percent of patients (*n* = 138) underwent 185 procedures on the duodeno‐pancreas or the stomach, but only 28% (*n* = 66) of procedures were intended to specifically treat ZES‐related gastrinomas, either for ZES only (*n* = 32; 17%) (ZES group), or for ZES associated with other pNETs (ZES + pNET group) (*n* = 34; 18%). Gastrointestinal surgery related to hyper acidity decreased dramatically (23%–6%), while surgery intended to cure ZES increased (20%–39%). Patients not operated for ZES were older at ZES diagnosis (46 vs. 42 years [*p* = 0.05]), were more frequently operated for another pNET during their life span (*p* < 0.001) (from 1 to 4 surgeries not related to gastrinomas), and were non‐significantly but more frequently metastatic at ZES diagnosis (6% vs. 3%). When considering both groups (ZES only and ZES + pNET), the use of a pancreaticoduodenectomy (PD) tended to increase slightly over time from 15% (*n* = 4) before 2000 to 35% (*n* = 14) thereafter (*p* = 0.09). All 66 patients had open procedures. Sixty‐four patients were classified pT1 while 2 were pT2. Otherwise, 30 patients were N1, 12 were N0, 24 were Nx and 4 were M1 [20].

**TABLE 1:** Patient characteristics (AFCE/GTE MEN1 cohort—MEN1‐related ZES study—1985–2015).

	Overall *n* = 233	Before 2000 *n* = 131	2000 and after *n* = 102	*p*
Male (*n* (%))	115 (49.4%)	65 (49.6%)	50 (49.0%)	0.900
Typical clinical ZES^a^ presentation (*n* (%))	162 (69.5%)	104 (79.4%)	58 (56.9%)	< 0.001
NIH diagnosis criteria of ZES^a^	148 (63.5%)	97 (74.0%)	51 (50.0%)	< 0.001
Gastrin level > 4.6 N^b^	91/187 (48.7%)	70/119 (58.8%)	21/68 (30.9%)	< 0.001
Age at MEN1 diagnosis (years) median (IQR^c^)	42.7 years (33.7–50.4)	42.8 years (35.6–50.7)	42.0 years (32.4–50.2)	0.475
Age at ZES^a^ diagnosis (years) median (IQR^c^)	43.6 years (35.5–53.1)	42.5 years (35.9–49.5)	45.4 years (35.1–56.5)	0.266
Overall rate of operated patients (stomach, duodenum, pancreas)	138 (59.2%)	86/131 (65.6%)	52/102 (51.0%)	0.020
Gastrointestinal surgery related to hyper acidity	36 (15.4%)	30 (22.9%)	6 (5.9%)	< 0.001
Percentage of patients operated for ZES	66 (28.3%)	26 (19.8%)	40 (39.2%)	0.001
Synchronous metastasis at ZES^a^ diagnosis	13 (5.6%)	9 (6.9%)	4 (3.9%)	0.400

^a^ ZES, Zollinger‐Ellison Syndrome.

^b^ N, Superior value of the normal range.

^c^ IQR, Inter Quartile Range.

### 3.2 Technical Choices in the ZES Group

The sequence of surgeries in the ZES group (i.e., without removal of associated pNET during the same surgery) are described in Table 2, which shows that duodenal‐focused surgery was the main technique used [(DUODX) (*n* = 16; 50%)], followed by pancreaticoduodenectomy [(PD) (*n* = 11; 34%)]. Seven different surgeries on the left pancreas were performed in five patients before duodenal surgery. PD resulted in two total pancreatectomies seeing as left pancreatectomies had already been performed in these cases. Table 3 provides more precise data related to the choice between PD and DUODX in the ZES group. Abnormal peri‐duodenal nodes were detected preoperatively on CT imaging in 80% of the PD group versus only 7% in the DUODX group (*p* < 0.001). Lymphadenectomies were carried out more frequently in the PD group [(PD) (*n* = 10/11; 90%)] [(DUODX) (*n* = 5/16; 31%)] (*p* < 0.01). The sensitivity of CT‐scan detection and the negative predictive value were both 100% (8/8 and 5/5).

**TABLE 2:** Overall surgeries among patients operated exclusively for ZES (*n* = 32) AFCE/GTE MEN1 cohort—MEN1‐related ZES study—(1985–2015).

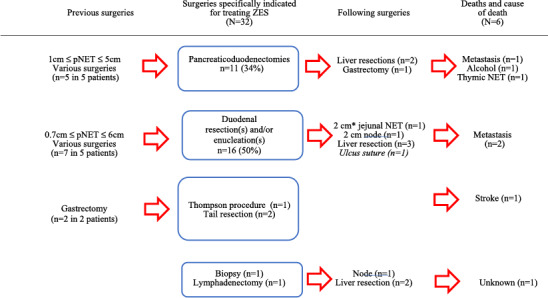

**TABLE 3:** Comparison of patients operated for ZES without associated pNET by DUODX and PD AFCE/GTE MEN1 cohort—MEN1‐related ZES study—1985–2015).

ID number	Procedure	Year of surgery	Severe GI disease	Previous procedure	Positive nodes (CTscan)	Invaded nodes (specimen)
338	PD	1993	Emergency	No	Not applicable	Not applicable
63	PD	1995	No	Tail resection	Negative	Negative
441	PD	1996	No	No	Positive	Positive
1269	PD	2002	Yes	No	Positive	Positive
96	PD	2003	No	Tail resection	Positive	Positive
116	PD	2006	Yes	No	Negative	Negative
333	PD	2007	Yes	Left pancreatectomy	Positive	Positive
336	PD	2010	No	Left pancreatectomy	Positive	Positive
661	PD	2013	No	No	Positive	Positive
647	PD	2013	No	Body resection	Positive	Positive
247	PD	2014	No	No	Positive	Negative
136	DUODX + gastrectomy + right hepatectomy	1986	Yes	No	Missing data	Positive
433	DUODX	1987	Yes	No	Negative	No lymphadenectomy
309	DUODX + antrectomy	1989	Yes	No	Negative	Negative
563	DUODX	1990	No	No	Missing data	No lymphadenectomy
317	DUODX	1992	Yes	No	Negative	No lymphadenectomy
473	DUODX	1993	Yes	No	Negative	No lymphadenectomy
687	DUODX	1993	Yes	Left pancreatectomy	Negative	No lymphadenectomy
7	DUODX	1996	No	Left pancreatectomy	Negative	No lymphadenectomy
220	DUODX	2000	No	Left pancreatectomy	Negative	No lymphadenectomy
55	DUODX	2001	No	Left pancreatectomy + head enucleation	Negative	No lymphadenectomy
1216	DUODX	2009	No	No	Positive	Positive
406	DUODX	2011	No	1‐Multiple enucleations	Negative	Negative
				2‐Head enucleation + tail resection		
1135	DUODX	2011	No	No	Negative	No lymphadenectomy
1129	DUODX	2013	Yes	No	Negative	No lymphadenectomy
1097	DUODX	2013	No	No	Negative	No lymphadenectomy
873	DUODX	2015	No	No	Negative	Negative

### 3.3 Technical Choices in the “ZES + pNET Group”

Details regarding the surgeries in the “ZES + pNET group” are provided in Table 4. Contrary to the “ZES” group, DUODX alone was never used since formal resections were required to remove at least one large associated pNET (≥ 2 cm) or rarely a secreting pNET (one insulinoma and one glucagonoma), depending on the anatomical location. Procedures removing both pNET(s) in the distal pancreas and duodenal gastrinomas (also called Thompson or Ann Arbor procedures) were used in 13 cases [22]. The insulinoma was removed using this technique. Total pancreatectomies (*n* = 9) were always used with the double indication of removing ZES‐related gastrinomas and other scattered large pNETs +/− associated with abnormal lymph nodes. The large glucagonoma was treated by total pancreatectomy. PD was used in order to fully remove duodenal ZES‐related gastrinomas and pNETs located in the head (*n* = 7). Eight surgical procedures had been performed previously (23%); four of them aimed to remove dp‐NETs. Among these cases, one patient was operated with distal pancreatectomy followed by PD, leading to total pancreatectomy.

**TABLE 4:** Indications and procedures among patients operated for ZES and associated pNETs (*n* = 34). AFCE/GTE MEN1 cohort—MEN1‐related ZES study—1985–2015.

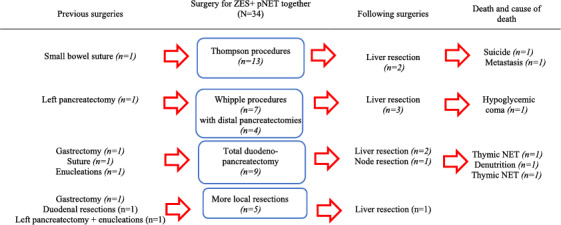

### 3.4 Lymphadenectomies During ZES Surgeries

Overall, associated lymphadenectomy was performed in 63% of patients operated for ZES (42/66), more frequently in cases of formal pancreatic resection (*n* = 36/45 [80%]) than in enucleations or more limited duodenal surgeries (*n* = 6/21 [30%]) (*p* < 0.001). Metastatic lymph nodes were found in 30/42 lymphadenectomies (71%). This percentage did not depend on surgical indication (ZES vs. ZES + pNET) (*p* = 0.7) nor on the type of surgery (formal pancreatic resection vs. other) (*p* = 0.15). Overall, associated pNETs may have been removed previously (*n* = 16/66 patients [24%]) or during ZES surgery (*n* = 34/66 patients [51%]). Therefore, it was not possible to determine whether the metastatic lymph nodes originated from a gastrinoma or from possible associated pNETs.

### 3.5 Survival and Causes of Death

**FIGURE 1:**
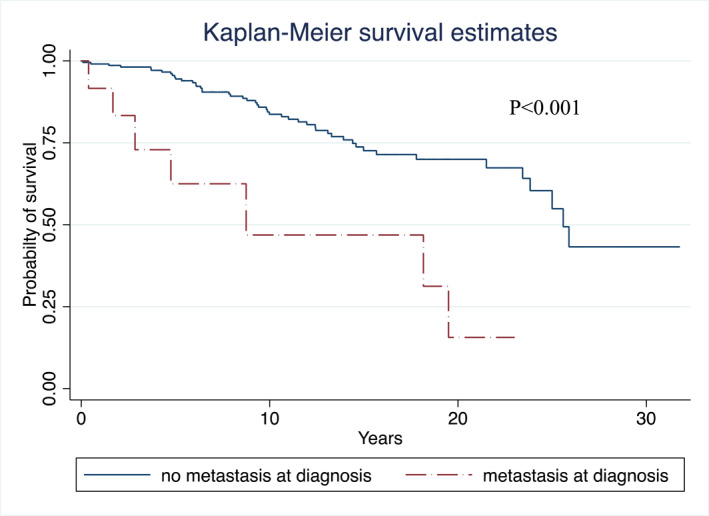
Overall survival of MEN1 related Zollinger‐Ellison patients. Metastatic versus non‐metastatic patients at Zollinger‐Ellison diagnosis. AFCE/GTE MEN1 cohort—MEN1‐related ZES study—1985–2015.

There were no perioperative deaths related to ZES surgery. Overall survival 15 years after ZES diagnosis was not significantly better among operated patients [82% (95% CI: 68–91) versus. 75% (95% CI: 58–85) (*p* = 0.2)]. Causes of death are displayed in Table 5. Of the 233 ZES patients, 13 (5.6%) presented distant metastases at diagnosis. Their overall survival was significantly decreased (Figure 1). Four of the 66 patients operated for ZES (6%) died as a result of metastatic duodeno‐pancreatic NETs versus 11 deaths in non‐operated patients (7%) (*p* = 1.0). Survival among operated patients was not associated with a pNET ≥ 2 cm removed during surgery (*p* = 0.7), the study period (*p* = 0.9), the indication (ZES vs. ZES + pNET) (*p* = 0.4), the removal of the head of the pancreas (*p* = 0.1), or the presence of invaded nodes (*p* = 0.3). Survival was higher among women than men (*p* = 0.04).

**TABLE 5:** Causes of death among operated and non‐operated patients (*n* = 55). AFCE/GTE MEN1 cohort—MEN1‐related ZES study—1985–2015).

*N* = 55		Related to DP‐NETs *N* = 21 (41.2%)			Non‐DP‐NET related cancers *N* = 15 (29.4%)			Other causes *N* = 15 (29.4%)	
4 Unknown causes *N* = 51		Operated patients	Non operated patients		Operated patients	Non operated patients		Operated patients	Non operated patients
Death related to MEN1 *N* = 31	dpNETs metastases	10 (19.6%)	5 (9.8%)	Thymic NET	4 (7.8%)	1 (1.9%)	Pulmonary embolism	1 (1.9%)	—
	Postoperative	1 (1.9%)	—	Bronchial NET	—	2 (3.9%)	Severe Cushing syndrome	—	1 (1.9%)
	Hypoglycemic coma	2 (3.9%)	—	Ependymoma	1 (1.9%)	—			
	Ulcerous disease	—	1 (1.9%)						
	Malnutrition	1 (1.9%)	—						
	Liver embolization	—	1 (1.9%)						
Death not related to MEN1 *N* = 20				Breast cancer^a^	2 (3.9%)	—	Stroke	2 (3.9%)	3 (5.9%)
				ENT cancer	2 (3.9%)	—	Acute cholangitis	1 (1.9%)	—
				Lung cancer	—	2 (3.9%)	Respiratory failure	—	2 (3.9%)
				Pancreatic cancer	—	1 (1.9%)	Alcohol	1 (1.9%)	—
							Suicide	1 (1.9%)	—
							Hip fracture	—	1 (1.9%)
							Gastroenteritis	1 (1.9%)	—
							Pancreatitis	1 (1.9%)	—

^a^ Breast cancers are possibly related to MEN1.

### 3.6 Effects of Surgery on Gastrin Levels

Gastrin levels were measured pre and post operatively in 49 patients with elevated gastrin levels. Normalization was observed in 61% (11/18) following removal of the duodenum and the head of the pancreas (PD or DPT). Normalization was observed in only 16% (5/31) of cases who underwent duodenum focused surgery (excisions or local duodenal resections) (*p* < 0.01). Two patients required additional surgery (one lymphadenectomy and one gastrinoma removal at the duodeno‐jejunal junction).

## 4 Discussion

While MEN1 disease is a rare condition, MEN1‐related ZES is even rarer. The present work is original in that it is the first to evaluate the “real‐life” surgical management of patients with MEN1‐related ZES/gastrinomas. We used this large nationwide MEN1 GTE‐AFCE cohort to evaluate the operative choices and their outcomes in the clinical context. Since MEN1 patients may be operated on for ZES and pNETs together, care was taken to distinguish indications for surgery depending on the presence or absence of large associated pNETs. This issue is important considering that large associated pNETs are likely to influence surgical strategy and prognosis. Nevertheless, the following findings warrant attention: (1) ZES presentation was less severe over time, but ZES diagnosis was not delayed despite of the lack of secreting tests. (2) Indications for ZES surgery increased over time. (3) Operative mortality was nil. (4) The surgical approach was highly dependent on the context, that is associated pNET(s), on abnormal nodes detected in the preoperative work‐up, or frequent previous pancreatic surgeries. (5) Metastatic lymph nodes were found in 71% of lymphadenectomies. (6) Fifteen‐year survival was slightly but non‐significantly better in patients operated for ZES versus non‐operated patients, but these two groups were not comparable regarding non‐ZES related surgeries and age at MEN1 diagnosis. (7) Patients with distant metastases at ZES diagnosis had significantly decreased survival. (8) Hypergastrinemia was normalized more effectively when the head of the pancreas and the duodenum were fully removed. Overall, the high rate of positive nodes, the decreased survival in patients with distant metastases, and the absence of perioperative mortality are relevant arguments when discussing the indication for surgery in MEN1‐ZES related patients. As far as technique is concerned, PD seems to be an appropriate option among fit patients. This study presents several limitations including a limited number of cases of operated ZES, a long inclusion period, the use of the UICC TNM classification versus the WHO classification (not available for all patients), the absence of description of surgical complications, and the absence quality of life evaluations. Moreover, and because they were not used throughout the study period, results of MRI and DOTATOC‐PET‐Gallium could not be analyzed in order to avoid a methodological bias.

### 4.1 Patient Characteristics and Changes Over Time

As expected, certain patient characteristics changed over time, especially those related to ZES diagnosis, potentially due to the increasing use of PPIs and the lack of secretin (Table 1) [19]. Age at MEN1 and ZES diagnosis did not change significantly. The use of PPIs explains the drop in surgery for hyperacidity. However, surgery for ZES increased dramatically (Table 1). Considering the debate about the interest of surgery for ZES considering the relatively good prognosis of ZES disease, this trend warrants further attention. The absence of operative mortality may have contributed to the increase in ZES surgery. When comparing survival between patients operated or not for ZES, two sources of bias should be noted: (1) ZES was diagnosed earlier in the operated arm, and (2) more duodenopancreatic surgeries were carried out in the “not operated for ZES” arm.

### 4.2 Technical Choices in the ZES Group

DUODX, which is focused on the duodenum, had low morbidity, no mortality, and was the most frequent surgery when no associated pNET had to be removed and no suspicious nodes were detected (Table 2). DUODX was clearly justified in five patients with previous pancreatic surgeries in order to spare the pancreatic parenchyma (15%). DUODX is also called the Thompson procedure when it is associated with a distal pancreatectomy and enucleation of any tumor in the pancreatic head or uncinate process [22]. However, the distal pancreatectomy is now less frequently used when there is no need to remove a large associated pNET in the distal part of the pancreatic gland. We know that the tumors located in the distal pancreas are usually not gastrinomas [23, 24]. Regarding the use of PD, suspicious nodes on preoperative CT imaging were key determinants favoring this technique in this ZES group (Table 3). PD associated with lymphadenectomy meets surgical oncologic criteria even if this procedure has major drawbacks and resulted in high operative mortality in the past. Previous nationwide studies from Germany and France found that 90‐day operative mortality after PD was 10.2% and 9.2%, respectively [25, 26]. Fortunately, operative mortality may be drastically reduced in high volume centers where failure‐to‐rescue is less frequent [27]. As mentioned above, PD operative mortality was nil in this series. Another study with no mortality from the Marburg group suggested that PD was associated with a better quality of life when compared to other surgical options [28]. Nevertheless, if a left resection has previously been performed, the use of PD is debatable in order to avoid total pancreaticoduodenectomy (TPD) (19% in this series). It also remains difficult to compare survival between PD and DUODX. Previous surgeries removed pNETs of up to 6 cm in 12 cases. Finally, the size of the ZES group is a limitation (Table 2).

### 4.3 Technical Choices in the “ZES + pNET Group”

Large associated pNETs may represent a surgical indication per se (Table 4). In patients presenting large pNETs, which were about half of cases, removing gastrinomas makes sense. The association of a resection of duodenal/pancreatic gastrinomas and a distal pancreatectomy (Thompson procedure) was used when the associated pNET was located in the left pancreas, and the Whipple procedure was used when the associated life‐threatening tumor was in the head. In the 1998 Thompson series, survival appeared to be better after surgery [22].

### 4.4 Lymphadenectomies During ZES Surgery

The fact that lymphadenectomies were more frequent in case of formal resections makes sense. Indeed, formal resections were used to treat cancer at least in the ZES + pNET group since pNETs were removed because of their risk of distant metastases. This oncologic purpose was also obvious in the ZES group when comparing the use of lymphadenectomy in case of DUODX versus PD. Table 3 shows that lymphadenectomies associated with PD were carried out when abnormal nodes were detected preoperatively. Clearly, surgeons choose more limited surgeries without lymphadenectomy in case of local disease and PD plus lymphadenectomy in the remainder of cases. PD enables an extensive lymphadenectomy and the complete removal of all duodenal gastrinomas. Abnormal nodes were detected by CT scan with good sensitivity and specificity even though CT was evaluated on a limited number of cases. Although MRI is now recommended for first‐line imaging, CT was the most commonly used imaging tool for localizing MEN1‐related dp‐NETs during the study period [4, 29].

### 4.5 Survival, Causes of Death and Impact of Surgery on Gastrinemia

Overall survival was not significantly better among operated patients. The 15‐year survival among non‐operated patients was quite good, confirming that gastrinomas are slow growing NETs. ZES disease has a longstanding spontaneous natural history. Therefore, randomized studies are very difficult to conduct, and indications for surgery must be considered cautiously. Moreover, this study highlights that associated pNETs operated on before or during ZES surgery represent a major prognosis bias. Fourteen out 66 patients (21.2%) had been operated on previously for one or several pNETs, which influences oncologic outcomes and technical aspects of management (Tables 2 and 4). This bias is also present in other published series, in which 67%–100% of ZES‐operated patients presented associated pNETs with sizes ranging from 0.2 to 3.7 cm [11, 28, 29]. Following the most recent international consensus, surgery is not absolutely contraindicated, but it does not seem justified in most cases seeing as no parameters have been shown to predict an aggressive course of disease [29]. Nevertheless, this prudent and understandable position may be readdressed for various reasons when considering present and past studies. (1) The GTE/AFCE and the NIH groups already confirmed that survival is decreased in MEN1 patients compared to the general population, mainly due to the metastatic evolution of dpNETs [6, 7, 23, 30]. (2) Metastatic patients at ZES diagnosis had very significantly decreased survival, and distant metastases accounted for 20% of deaths (Figure 1 and Table 5). (3) Another previous AFCE/GTE study, using a Markov model, showed that large pNETs (≥ 2 cm, functional or not) but mostly gastrinomas (whatever their size) were both independently and significantly responsible for the occurrence of distant metastases among MEN1 patients (hazard ratio: 2.96 [1.43–6.14] and 4.36 [2.44–6.14] respectively) [9]. (4) The present study also found that 71% of lymphadenectomies were already positive after ZES surgery among selected patients. Indeed, lymph node involvement around the pancreas, whatever the origin, is the first step toward distant metastatic spread. (5) In an epidemiological study involving 7613 patients with non‐MEN1 duodenal neuroendocrine tumors, the authors found that the presence of more than nine positive lymph nodes was significantly associated with decreased overall survival while the surgical technique had no influence (i.e., local vs. anatomic surgery) [31]. (6) In another large epidemiological study including 5502 patients with sporadic duodenal neuroendocrine tumors, local or anatomic resection was associated with increased overall survival [32]. (7) Operative mortality was nil in the population presented here, similar to other series [28, 29, 33, 34, 35]. This is in line with the fact that pancreatic surgery has become safer when performed in specialized centers. (8) More recently, Imamura, using an unusual surgical technique selectively removing the duodenum with lymphadenectomy, showed better survival and a drop in gastrinemia in operated patients versus those who were only monitored [33]. Similar to the results of the Marburg group, this study observed better control of gastrinemia following PD [28, 35]. Finally, it is worth keeping in mind that the indication for surgery in case of pNETs larger than 2 cm has already been validated with the same justifications as those presented here for ZES. In both situations, the rationale was based on high risk of lymph node involvement and of distant metastasis occurrence. Nevertheless, there are many reasons that could explain why surgery was not carried out (72% of the cohort). First, even if the surgical indication seems reasonable, it is important to assess the presence of other MEN1‐related tumors such as thNETs, brNETs, and ependymomas, which were responsible here for 16% of deaths (Table 2). This explanation was also mentioned by Norton and R.T. Jensen [36]. Moreover, patients were more frequently older, with a more complicated surgical history. Synchronous metastases were found non‐significantly but more frequently at ZES diagnosis. Logically, indications for surgery were less frequent in the earlier part of the study when pancreatic surgery was not as safe. Finally, considering that MEN1‐patients have a 50% probability of being operated on three times for various MEN1 tumors during their lifetime, they may also be unfavorable to the prospect of yet another surgery [30].

## 5 Conclusion

**FIGURE 2:**
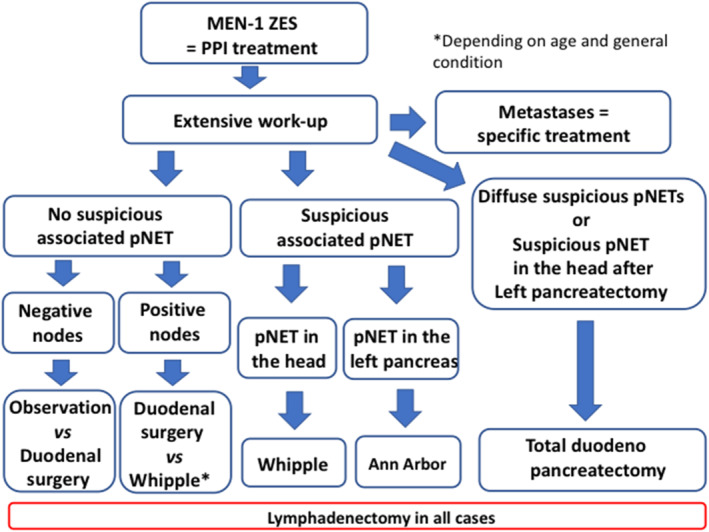
Surgical decision‐making flowchart. AFCE/GTE MEN1 cohort—MEN1‐related ZES study—1985–2015.

This multicenter observational study reflects real‐life practice for MEN1‐related ZES surgery. In the absence of randomized studies, this evidence‐based cohort analysis suggests that surgery for “MEN1‐related ZES” may be offered safely in selected cases. This study also shows that the surgical technique must be discussed on a case‐by‐case basis in specialized centers given the high numbers of factors that have to be taken in account (Figure 2). Operating on ZES in fit MEN1 patients may avoid metastatic spread and improve metastasis‐free survival. This means that the patient is more likely to avoid cancer treatment and enjoy a better quality of life, which is a key consideration for the management of patients with MEN1 NETs. Overall, these results should provide physicians with relevant data as they continue to discuss further recommendations for MEN1 patients with ZES. In addition, new techniques such as DOTATOC‐PET‐Gallium, submucosal resections, and molecular analysis of the lymph nodes are now available and can be used to help support the decision‐making process.

## Author Contributions

**Sébastien Gaujoux:** conceptualization, methodology, supervision, validation, writing – review and editing. **François Pattou:** conceptualization, validation. **Guillaume Cadiot:** supervision, validation. **Mustapha Adham:** supervision, validation. **Philippe Bachellier:** supervision, validation. **Jean‐Pierre Bail:** supervision, validation. **Robert Caiazzo:** supervision, validation. **Nicolas Carrere:** supervision, validation. **Philippe Chaffanjon:** supervision, validation. **Sophie Deguelte:** supervision, validation. **Gianluca Donatini:** supervision, validation. **Bertrand Dousset:** supervision, validation. **Matthieu Faron:** supervision, validation. **Caroline Gronnier:** supervision, validation. **Bruno Heyd:** supervision, validation. **Jean‐Christophe Lifante:** supervision, validation. **Jean Lubrano:** supervision, validation. **Nicolas Meurisse:** supervision, validation. **Eric Mirallié:** supervision, validation. **Nicolas Santucci:** supervision, validation. **Alain Sauvanet:** supervision, validation. **Frédéric Sebag:** supervision, validation. **Laurent Sulpice:** validation, visualization, supervision. **Baudoin Thebault:** supervision, validation. **Jean‐Jacques Tuech:** supervision, validation. **Thomas Walter:** supervision, validation. **Christine Binquet:** conceptualization, supervision, software, validation, writing – original draft. **Pierre Goudet:** conceptualization, data curation, formal analysis, investigation, methodology, project administration, validation, writing – original draft, writing – review and editing.

## Conflicts of Interest

The authors declare no conflicts of interest.

## Supporting information


Supporting Information S1

## Data Availability

The data that support the findings of this study are available from the corresponding author upon reasonable request.
