# Supplementary material for: Surgical Management of Zollinger‐Ellison Syndrome in Multiple Endocrine Neoplasia Type 1 an AFCE and GTE Cohort Study. (Association Francophone de Chirurgie Endocrinienne and Groupe d'étude des Tumeurs Endocrines)
Source: World J Surg. 2026 Mar 20;50(5):1300–11. doi: 10.1002/wjs.70303 (PMC13206476; doi:10.1002/wjs.70303)
Supplement: Supplementary file 1 — Supporting Information S1 [file WJS-50-1300-s001.docx]

Supplemental material:

1°) The French national cohort

MEN1 cases were detected both by the French network of genetic laboratories in charge of the diagnosis and by the national reference centers for Neuro Endocrine Tumors where the multidisciplinary decisions are made (RENATEN; REseau NAtional de référence pour la prise en charge des Tumeurs neuro ENdocrines; (https://www.reseau-gte.org/renaten/https://www.reseau-gte.org/renaten/). Copies of clinical, surgical and pathological reports were obtained. Variable of interest are computerized using a 367-item form. All data was controlled by a specialized clinician (PG) and collected in a secure database at the Dijon Clinical Investigation Centre (Dijon-Bourgogne University Hospital and INSERM - Institut National de la Santé et de la Recherche Médicale - CIC1432) and monitored regularly. The MEN-1 cohort was approved by the CCTIRS (Comité Consultatif sur le Traitement de l'Information en matière de Recherche - Consultative Committee on Treatment of Information in Health Research, file number 12.364) and the CNIL (Commission Nationale de l'Informatique et des Libertés - National Committee for Data Protection, authorization number DR 2013-348) including an authorization to contact General Practitioners and patients (n°912352).

2°) Criteria for the diagnosis of MEN1 (Summary)

The criteria include 1) patients with at least one lesion of the MEN1 spectrum and a MEN-1 mutation, 2) patients belonging to an identified MEN-1 family in which at least one first-degree relative had been affected and who had at least one MEN-1-related lesion, and 3) patients without positive genetic testing or family history of the disease, but with at least two of the three major MEN-1 lesions (pHPT, PD-NETs and pituitary tumors). Patients meeting the third inclusion criteria were included with caution since these associations may randomly occur in the general population. In such cases, additional criteria were used to help with the diagnosis: known or suggestive family history, lesions occurring before age 35, presence of pituitary macroadenoma, multiglandular pHPT, pHPT relapsing after surgery, multiple neuroendocrine tumors within the pancreas, and presence of other MEN1-related lesions such as adrenal NETs, thymic NETs (th-NETs), bronchial NETS (br-NETs), brain tumors or skin lesions.

3°) Criteria for ZES diagnosis and results.

Patients with at least 2 positive NIH criteria were diagnosed as ZES whatever the clinical presentation of their duodeno-gastric disease. By contrast, patients with only one NIH criteria associated to ulcerous disease were diagnosed as ZES on endoscopical and clinical grounds ascertained by the Gastro-enterologist. One-hundred-and-forty-eight cases (63%) fulfilled at least 2 NIH criteria. Eighty-five cases (37%) presented only 1 out 5 NIH criteria (**Table 1**).

Table 1

Details of diagnosis criteria of 233 ZES patients.

| ID | Year of ZES diagnosis | At least 2 NIH criteria | Clinical and endoscopical ZES + 1 NIH criteria | Gastrin (without PPI) | Gastrin + secretin test | Basal acid output | Secretin Stimulated acid output | Fine Needle Aspiration (FNA) or biopsy/histology |
| --- | --- | --- | --- | --- | --- | --- | --- | --- |
| 2 | 1985 | yes | no | elevated | not done | elevated | not done | no |
| 32 | 1985 | yes | No | elevated | elevated | elevated | elevated | no |
| 100 | 1985 | yes | no | elevated | not available | not done | not done | yes |
| 106 | 1985 | yes | No | elevated | elevated | not done | not done | yes |
| 132 | 1985 | yes | No | elevated | elevated | elevated | elevated | no |
| 473 | 1985 | yes | no | elevated | not available | not done | not done | yes |
| 503 | 1985 | no | yes | elevated | not done | not done | not done | no |
| 987 | 1985 | yes | No | elevated | elevated | elevated | not done | no |
| 998 | 1985 | no | yes | elevated | not available | not done | not done | no |
| 40 | 1986 | yes | No | elevated | elevated | elevated | Normal | no |
| 63 | 1986 | no | yes | not available | not available | not available | not available | yes |
| 71 | 1986 | yes | No | elevated | elevated | elevated | elevated | no |
| 115 | 1986 | yes | no | elevated | not available | not done | not done | yes |
| 136 | 1986 | yes | No | elevated | elevated | not done | not done | yes |
| 231 | 1986 | no | yes | elevated | not available | not done | not done | no |
| 20 | 1987 | yes | No | elevated | elevated | Normal | elevated | no |
| 69 | 1987 | yes | No | elevated | elevated | Normal | elevated | no |
| 117 | 1987 | yes | No | elevated | elevated | not done | not done | yes |
| 120 | 1987 | yes | No | elevated | elevated | elevated | Normal | no |
| 156 | 1987 | no | yes | elevated | not done | not done | not done | no |
| 183 | 1987 | yes | no | elevated | not done | not done | not done | yes |
| 210 | 1987 | no | yes | elevated | not done | not done | not done | no |
| 433 | 1987 | no | yes | elevated | not available | not done | not done | no |
| 540 | 1987 | yes | No | elevated | not available | elevated | not done | no |
| 84 | 1988 | no | yes | elevated | not done | not done | not done | no |
| 114 | 1988 | yes | no | elevated | not done | elevated | not done | no |
| 123 | 1988 | yes | No | elevated | elevated | elevated | elevated | yes |
| 248 | 1988 | no | yes | elevated | not done | not done | not done | no |
| 49 | 1989 | yes | No | elevated | elevated | elevated | not done | no |
| 83 | 1989 | yes | no | not available | not available | elevated | elevated | no |
| 111 | 1989 | yes | No | elevated | elevated | elevated | not done | no |
| 309 | 1989 | yes | No | elevated | not available | Normal | not done | yes |
| 317 | 1989 | yes | No | elevated | elevated | not done | not done | yes |
| 335 | 1989 | yes | No | elevated | elevated | elevated | not done | yes |
| 417 | 1989 | yes | no | elevated | not done | not done | not done | yes |
| 434 | 1989 | yes | No | elevated | elevated | elevated | elevated | no |
| 65 | 1990 | yes | No | elevated | elevated | elevated | not done | yes |
| 119 | 1990 | yes | no | elevated | not available | not done | not done | yes |
| 191 | 1990 | yes | No | elevated | elevated | elevated | elevated | no |
| 223 | 1990 | no | yes | elevated | not done | not done | not done | no |
| 280 | 1990 | yes | No | elevated | elevated | elevated | Normal | no |
| 300 | 1990 | yes | No | elevated | elevated | elevated | elevated | no |
| 361 | 1990 | yes | No | elevated | elevated | Normal | elevated | no |
| 424 | 1990 | yes | no | elevated | not available | not done | not done | yes |
| 548 | 1990 | yes | No | elevated | elevated | elevated | elevated | no |
| 563 | 1990 | no | yes | elevated | not available | not done | not done | no |
| 893 | 1990 | no | yes | elevated | not done | not done | not done | no |
| 1148 | 1990 | yes | no | elevated | not done | not done | not done | yes |
| 1301 | 1990 | no | yes | elevated | not done | not done | not done | no |
| 8 | 1991 | no | yes | elevated | not done | not done | not done | no |
| 51 | 1991 | yes | No | elevated | not available | elevated | not done | yes |
| 99 | 1991 | yes | No | elevated | elevated | not done | not done | no |
| 112 | 1991 | yes | No | elevated | not available | elevated | not done | no |
| 147 | 1991 | no | yes | elevated | not done | not done | not done | no |
| 220 | 1991 | yes | No | elevated | elevated | not done | not done | yes |
| 367 | 1991 | yes | No | elevated | not available | elevated | elevated | no |
| 541 | 1991 | yes | No | elevated | elevated | elevated | elevated | yes |
| 549 | 1991 | yes | No | elevated | not available | elevated | elevated | yes |
| 676 | 1991 | no | yes | Normal | elevated | not done | not done | no |
| 116 | 1992 | no | yes | Normal | elevated | Normal | not done | no |
| 150 | 1992 | no | yes | elevated | not done | not done | not done | no |
| 336 | 1992 | yes | yes | Normal | elevated | Normal | Normal | yes |
| 400 | 1992 | yes | No | elevated | elevated | elevated | elevated | yes |
| 443 | 1992 | yes | No | elevated | elevated | not done | not done | no |
| 500 | 1992 | yes | No | elevated | not available | elevated | elevated | no |
| 687 | 1992 | no | yes | elevated | not done | not done | not done | no |
| 198 | 1993 | yes | No | elevated | elevated | not done | not done | yes |
| 338 | 1993 | no | yes | elevated | not available | not done | not done | no |
| 546 | 1993 | yes | No | elevated | elevated | elevated | not done | no |
| 752 | 1993 | yes | No | elevated | elevated | not done | not done | no |
| 769 | 1993 | yes | no | elevated | not done | not done | not done | yes |
| 87 | 1994 | yes | No | elevated | not available | elevated | not done | no |
| 141 | 1994 | yes | No | elevated | elevated | elevated | not done | no |
| 161 | 1994 | yes | No | elevated | not available | elevated | elevated | yes |
| 162 | 1994 | no | yes | elevated | not done | not done | not done | no |
| 164 | 1994 | yes | No | elevated | elevated | elevated | not done | yes |
| 221 | 1994 | yes | No | elevated | not available | elevated | elevated | no |
| 270 | 1994 | yes | No | elevated | elevated | elevated | elevated | no |
| 493 | 1994 | yes | No | elevated | elevated | Normal | elevated | no |
| 513 | 1994 | yes | No | elevated | not available | Normal | elevated | no |
| 834 | 1994 | no | yes | elevated | not available | not done | not done | no |
| 874 | 1994 | yes | no | elevated | not done | not done | not done | yes |
| 104 | 1995 | yes | No | elevated | elevated | elevated | elevated | no |
| 190 | 1995 | yes | No | elevated | elevated | elevated | elevated | yes |
| 197 | 1995 | no | yes | elevated | not available | not done | not done | no |
| 282 | 1995 | yes | No | elevated | elevated | not done | not done | no |
| 330 | 1995 | yes | No | elevated | elevated | not done | not done | no |
| 362 | 1995 | yes | No | elevated | elevated | elevated | elevated | no |
| 441 | 1995 | yes | No | elevated | elevated | elevated | elevated | yes |
| 447 | 1995 | yes | No | elevated | not available | elevated | elevated | no |
| 7 | 1996 | yes | no | elevated | not done | not done | not done | yes |
| 380 | 1996 | yes | no | elevated | not done | not done | not done | yes |
| 391 | 1996 | no | yes | elevated | not done | not done | not done | no |
| 396 | 1996 | yes | No | elevated | elevated | not done | not done | no |
| 899 | 1996 | no | yes | elevated | not available | not done | not done | no |
| 55 | 1997 | yes | no | Normal | elevated | elevated | elevated | yes |
| 160 | 1997 | yes | no | elevated | not available | not done | not done | yes |
| 254 | 1997 | yes | no | elevated | not available | not done | not done | yes |
| 264 | 1997 | yes | No | elevated | elevated | not done | not done | no |
| 307 | 1997 | no | yes | elevated | not done | not done | not done | no |
| 377 | 1997 | yes | No | elevated | elevated | not done | not done | no |
| 397 | 1997 | yes | no | elevated | not done | not done | not done | yes |
| 419 | 1997 | yes | No | elevated | not available | elevated | elevated | no |
| 442 | 1997 | no | yes | elevated | not done | not done | not done | no |
| 547 | 1997 | no | yes | elevated | not available | not done | not done | no |
| 742 | 1997 | no | yes | elevated | not done | not done | not done | no |
| 801 | 1997 | yes | No | elevated | elevated | Normal | not done | no |
| 52 | 1998 | yes | no | elevated | not available | not done | not done | yes |
| 212 | 1998 | yes | No | elevated | elevated | elevated | elevated | no |
| 304 | 1998 | yes | No | elevated | elevated | not done | not done | yes |
| 429 | 1998 | no | yes | elevated | not done | not done | not done | no |
| 444 | 1998 | yes | no | elevated | not done | not done | not done | yes |
| 481 | 1998 | yes | No | elevated | elevated | not done | not done | no |
| 542 | 1998 | yes | No | elevated | elevated | Normal | Normal | no |
| 552 | 1998 | yes | no | Normal | elevated | not done | not done | yes |
| 932 | 1998 | no | yes | elevated | not available | not done | not done | no |
| 214 | 1999 | yes | No | elevated | not available | elevated | not done | no |
| 263 | 1999 | yes | No | elevated | elevated | not done | not done | no |
| 295 | 1999 | yes | No | elevated | elevated | not done | not done | yes |
| 323 | 1999 | yes | no | elevated | not done | not done | not done | yes |
| 386 | 1999 | no | yes | elevated | not done | not done | not done | no |
| 458 | 1999 | yes | no | elevated | not done | not done | not done | yes |
| 492 | 1999 | yes | No | elevated | elevated | elevated | elevated | yes |
| 504 | 1999 | yes | No | elevated | elevated | elevated | elevated | no |
| 526 | 1999 | yes | No | elevated | elevated | not done | not done | yes |
| 553 | 1999 | yes | No | elevated | elevated | not done | not done | yes |
| 578 | 1999 | yes | No | elevated | elevated | not done | not done | yes |
| 595 | 1999 | yes | no | elevated | not available | not done | not done | yes |
| 733 | 1999 | yes | no | elevated | not available | not done | not done | yes |
| 773 | 1999 | no | yes | elevated | not done | not done | not done | no |
| 785 | 1999 | no | yes | elevated | not available | not done | not done | no |
| 73 | 2000 | yes | No | elevated | elevated | elevated | elevated | no |
| 159 | 2000 | no | yes | Normal | not available | Normal | elevated | no |
| 209 | 2000 | no | yes | elevated | not done | not done | not done | no |
| 545 | 2000 | no | yes | elevated | not done | not done | not done | no |
| 686 | 2000 | yes | no | elevated | not done | not done | not done | yes |
| 688 | 2000 | no | yes | elevated | not done | not done | not done | no |
| 698 | 2000 | no | yes | elevated | not done | not done | not done | no |
| 710 | 2000 | yes | No | elevated | elevated | elevated | Normal | no |
| 753 | 2000 | yes | no | elevated | not done | not done | not done | yes |
| 824 | 2000 | yes | no | elevated | not done | not done | not done | yes |
| 833 | 2000 | yes | no | elevated | not done | not done | not done | yes |
| 900 | 2000 | no | yes | elevated | not done | not done | not done | no |
| 931 | 2000 | yes | no | elevated | not available | not done | not done | yes |
| 1004 | 2000 | no | yes | elevated | not done | not done | not done | no |
| 495 | 2001 | yes | no | elevated | not done | not done | not done | yes |
| 539 | 2001 | no | yes | Normal | elevated | Normal | Normal | no |
| 714 | 2001 | yes | No | elevated | elevated | not done | not done | yes |
| 755 | 2001 | yes | No | elevated | elevated | not done | not done | no |
| 845 | 2001 | no | yes | elevated | not available | not done | not done | no |
| 1037 | 2001 | yes | No | elevated | elevated | Normal | elevated | no |
| 1218 | 2001 | yes | No | elevated | elevated | elevated | elevated | yes |
| 226 | 2002 | yes | No | elevated | elevated | Normal | elevated | yes |
| 325 | 2002 | no | yes | elevated | not done | not done | not done | no |
| 675 | 2002 | no | yes | elevated | not done | not done | not done | no |
| 691 | 2002 | no | yes | elevated | not done | not done | not done | no |
| 760 | 2002 | yes | no | elevated | not available | not done | not done | yes |
| 1053 | 2002 | no | yes | elevated | not done | not done | not done | no |
| 1069 | 2002 | no | yes | elevated | not done | not done | not done | no |
| 1269 | 2002 | no | yes | elevated | not available | not done | not done | no |
| 96 | 2003 | yes | no | elevated | not available | not done | not done | yes |
| 457 | 2003 | yes | No | elevated | elevated | not done | not done | no |
| 577 | 2003 | no | yes | elevated | not available | not done | not done | no |
| 974 | 2003 | yes | no | elevated | not done | not done | not done | yes |
| 1152 | 2003 | yes | no | elevated | not done | not done | not done | yes |
| 279 | 2004 | yes | No | elevated | elevated | Normale | elevated | no |
| 647 | 2004 | yes | No | elevated | not available | Normal | Normal | yes |
| 728 | 2004 | yes | No | elevated | elevated | elevated | elevated | no |
| 997 | 2004 | yes | No | elevated | not available | Normal | elevated | no |
| 1058 | 2004 | no | yes | elevated | not available | not done | not done | no |
| 1120 | 2004 | no | yes | elevated | not done | not done | not done | no |
| 550 | 2005 | no | yes | elevated | not available | not done | not done | no |
| 764 | 2005 | no | yes | elevated | not done | not done | not done | no |
| 780 | 2005 | no | yes | elevated | not available | not done | not done | no |
| 796 | 2005 | no | yes | elevated | not done | not done | not done | no |
| 1129 | 2005 | no | yes | elevated | not available | not done | not done | no |
| 1158 | 2005 | yes | no | elevated | not available | not done | not done | yes |
| 1163 | 2005 | yes | No | elevated | elevated | elevated | elevated | no |
| 1324 | 2005 | no | yes | elevated | not done | not done | not done | no |
| 333 | 2006 | yes | no | elevated | not available | not done | not done | yes |
| 372 | 2006 | yes | No | elevated | elevated | Normal | Normal | yes |
| 977 | 2006 | no | yes | elevated | not available | not done | not done | no |
| 1005 | 2006 | no | yes | elevated | not done | not done | not done | no |
| 1060 | 2006 | no | yes | elevated | not done | not done | not done | no |
| 1135 | 2006 | yes | No | elevated | elevated | not done | not done | yes |
| 1213 | 2006 | yes | no | elevated | not done | not done | not done | yes |
| 1312 | 2006 | no | yes | elevated | not done | not done | not done | no |
| 1344 | 2006 | no | yes | elevated | not done | not done | not done | no |
| 205 | 2007 | yes | no | elevated | not done | Normal | not done | yes |
| 247 | 2007 | no | yes | Normal | not available | Normal | elevated | no |
| 420 | 2007 | yes | No | Normal | elevated | not done | not done | yes |
| 692 | 2007 | yes | No | elevated | elevated | not done | not done | no |
| 1036 | 2007 | no | yes | elevated | not available | not done | not done | no |
| 1047 | 2007 | yes | No | elevated | elevated | elevated | elevated | no |
| 1049 | 2007 | yes | No | elevated | elevated | Normal | Normal | yes |
| 1066 | 2007 | no | yes | elevated | not done | not done | not done | no |
| 1097 | 2007 | yes | No | elevated | not available | elevated | not done | yes |
| 1200 | 2007 | no | yes | elevated | not available | not done | not done | no |
| 174 | 2008 | no | yes | elevated | not done | not done | not done | no |
| 421 | 2008 | no | yes | elevated | not available | not done | not done | no |
| 470 | 2008 | yes | No | elevated | elevated | not done | not done | no |
| 1020 | 2008 | no | yes | elevated | not done | not done | not done | no |
| 1141 | 2008 | no | yes | elevated | not available | not done | not done | no |
| 1208 | 2008 | no | yes | elevated | not available | not done | not done | no |
| 1209 | 2008 | yes | no | elevated | not done | not done | not done | yes |
| 1216 | 2008 | yes | no | elevated | not done | not done | not done | yes |
| 1320 | 2008 | no | yes | elevated | not done | not done | not done | no |
| 1357 | 2008 | no | yes | elevated | not done | not done | not done | no |
| 496 | 2009 | yes | no | elevated | not done | not done | not done | yes |
| 873 | 2009 | no | yes | elevated | not done | not done | not done | no |
| 1026 | 2009 | yes | no | elevated | not done | elevated | not done | no |
| 841 | 2010 | no | yes | elevated | not done | not done | not done | no |
| 1008 | 2010 | no | yes | elevated | not done | not done | Normal | no |
| 1106 | 2010 | yes | no | elevated | not available | not done | not done | yes |
| 216 | 2011 | yes | no | elevated | not done | elevated | not done | no |
| 225 | 2011 | yes | no | elevated | not done | elevated | not done | no |
| 258 | 2011 | yes | no | elevated | not done | not done | not done | yes |
| 334 | 2011 | no | yes | elevated | not available | not available | not available | no |
| 406 | 2011 | yes | No | elevated | elevated | elevated | elevated | yes |
| 666 | 2011 | no | yes | elevated | not done | not done | not done | no |
| 1178 | 2011 | yes | no | elevated | not done | elevated | not done | no |
| 1283 | 2011 | no | yes | elevated | not available | not done | not done | no |
| 1358 | 2011 | no | yes | elevated | not done | not done | not done | no |
| 1359 | 2011 | yes | no | elevated | not done | not done | not done | yes |
| 602 | 2012 | no | yes | elevated | not done | not done | not done | no |
| 661 | 2012 | yes | no | elevated | not available | not done | not done | yes |
| 1245 | 2012 | no | yes | elevated | not available | not done | not done | no |
| 1256 | 2012 | yes | no | elevated | not done | not done | not done | yes |
| 1276 | 2013 | yes | no | elevated | not done | not done | not done | yes |
| 1305 | 2013 | yes | no | elevated | not done | not done | not done | yes |
| 271 | 2014 | yes | No | elevated | elevated | Normal | Normal | no |
| 1243 | 2014 | no | yes | elevated | not done | not done | not done | no |
| 1285 | 2015 | no | yes | elevated | not done | not done | not done | no |

|  |  |  |  |  |  |  |  |  |
| --- | --- | --- | --- | --- | --- | --- | --- | --- |
